# Quality Measure Adherence and Oral Health Outcomes in Children

**DOI:** 10.1001/jamanetworkopen.2023.53861

**Published:** 2024-01-30

**Authors:** Sung Eun Choi, Ankur Pandya, Joel White, Elizabeth Mertz, Sharon-Lise Normand

**Affiliations:** 1 Department of Oral Health Policy and Epidemiology, Harvard School of Dental Medicine, Boston, Massachusetts; 2 Department of Health Policy and Management, Harvard T. H. Chan School of Public Health, Boston, Massachusetts; 3 Department of Preventive and Restorative Dental Sciences, School of Dentistry, University of California, San Francisco; 4 Department of Health Care Policy, Harvard Medical School, Boston, Massachusetts; 5 Department of Biostatistics, Harvard T. H. Chan School of Public Health, Boston, Massachusetts

## Abstract

**Importance:**

Process-based quality measures are generally intended to promote evidence-based practices that have been proven to improve outcomes. However, due to lack of standardized implementation of diagnostic codes in dentistry, assessing the association between process and oral health outcomes has been challenging.

**Objective:**

To estimate the association of adhering to dental quality measures with patient oral health outcomes.

**Design, Setting, and Participants:**

Using a target trial emulation, a causal inference framework, this retrospective cohort study estimated the difference in the risk of developing tooth decay between US children who adhered to process-based dental quality measures (receiving topical fluoride and sealant [treated groups]) and those who did not (control groups). Electronic health records of US children and adolescents aged 0 to 18 years from January 1, 2014, to December 31, 2020, were used. To emulate random treatment assignment based on baseline confounders, coarsened exact matching was used to produce covariate balance between the treated and control groups. A time-to-event regression model produced effect estimates, adjusting for time-varying covariates. Near-far matching was used to account for unmeasured confounders as a sensitivity analysis. Data were analyzed from May 1 to August 7, 2023.

**Exposures:**

Adherence to dental quality measures.

**Main Outcomes and Measures:**

Incidence of tooth decay.

**Results:**

Among 69 212 US children aged between 0 and 18 years (mean [SD] age, 10.2 [5.0] years; 49.5% male, 50.4% female, and 0.1% unknown or transgender), 1930 (2.8%) were Asian, 2038 (2.9%) were Black, 8667 (12.5%) were Hispanic, 33 632 (48.6%) were White, and 22 945 (33.2%) were multiracial, other, or missing racial and ethnic group identification. Relative to control individuals, treated individuals were more likely to be at elevated risk of caries (fluoride measure: 16 453 [76.5%] vs 15 236 [39.8%]; sealant measure: 2264 [54.6%] vs 997 [44.0%]) and have regular dental visits (fluoride measure: 21 498 [100%] vs 13 741 [35.9%]; sealant measure: 1623 [39.2%] vs 871 [38.4%]). Adherence to quality measures was associated with reduced risk of tooth decay with adjusted hazard ratios of 0.82 (95% CI, 0.78- 0.86) for fluoride and 0.86 (95% CI, 0.76-0.97) for sealant in the matched cohort. Benefits of adhering to quality measures were greater among children at elevated vs low risk and with public vs commercial insurance for both measures.

**Conclusions:**

In this cohort study, adhering to dental quality measures was associated with reduced risk of tooth decay, and benefits were greater among children at elevated risk and with public insurance. These findings provide insights in facilitating targeted application of quality measures or developing more tailored quality improvement initiatives.

## Key Points

**Question:**

What is the association of adhering to process-based dental quality measures with oral health outcomes among US children?

**Findings:**

This cohort study of 69 212 children and adolescents found that adhering to dental quality measures (topical fluoride application and sealant receipt) was associated with reduced risk of tooth decay. Benefits of adhering to these quality measures were greater among children at elevated vs low risk and with public vs commercial insurance.

**Meaning:**

Supporting application of fluoride and sealant in nondental settings or by primary care clinicians could increase access to recommended dental care and thereby improve children’s oral health.

## Teaser

This cohort study evaluates whether adherence to pediatric oral health quality measures is associated with improved patient oral health outcomes among US children.

## Introduction

Health care decision-makers have explicit and implicit incentives to improve the quality of care delivered in the US.^1,2^ Since the Children’s Health Insurance Program Reauthorization Act of 2009 established the child core set to strengthen the quality of health care provided to children enrolled in Medicaid,^3^ the mandatory reporting of the child core set of health care quality measures beginning in 2024 would be a major step forward toward quality improvements.^4^ Quality measures are intended to increase quality of care by establishing a more transparent, evidence-informed, and person-centered care system.^5^ While several conceptual frameworks exist to define quality of care, the Institute of Medicine has defined health care quality as “the degree to which health services for individuals and populations increase the likelihood of desired health outcomes and are consistent with current professional knowledge.”^6^ This approach incorporates 2 elements in measuring health care quality: (1) effects of health care on health outcomes and (2) the degree to which health care adheres to processes.^7,8^ However, many quality measures that are currently used are process-based measures, as they are relatively easy to measure and responsive in pay-for-performance systems. To be valid process-based measures, the results of the process need to improve patient health outcomes.

The Institute of Medicine identified underuse of quality measures in dentistry, many of which were process-based (eg, topical fluoride application) and developed over a decade ago, as a barrier to quality improvement in oral health care.^9,10,11,12^ Research regarding dental quality assessment and its effects has several challenges. First, quality assessment for oral health is limited by the absence of universally accepted and used diagnostic codes for outcomes in dentistry. By focusing on procedure codes instead, dental records and billing systems capture the number of dental procedures performed but provide little insight on how these procedures affect the oral health status of the patients. Another issue is related to the generalizability of randomized clinical trials (RCTs). While several RCTs have evaluated the efficacy of preventive dental care on oral health outcomes, many of these trials were school-based programs, targeting specific subgroups of the population.^13,14,15,16^ Moreover, substantial heterogeneities were observed among these RCT studies.^17,18^

Target trial emulation (TTE), a framework for causal inference using observational data, may help address these issues in dental quality assessment by emulating a hypothetical randomized trial.^19^ Using observational data that contain oral health records, TTE can help to estimate the association between adherence to dental quality measures and oral health outcomes among broader populations (eligible population for the measure’s inclusion requirements) than those included in RCTs. Due to the lack of standardized implementation of diagnostic codes in dentistry, little has been known of the links between quality measures and patient oral health outcomes limiting adaptation of value-based oral health care. We sought to evaluate whether adherence to pediatric oral health quality measures is associated with improved patient oral health outcomes among US children.

## Methods

### Study Population

Using deidentified electronic health records (EHR) data of children and adolescents who have enrolled in and received care through a large dental accountable care organization with more than 50 dental offices in Washington, Oregon, and Idaho, this cohort study included 69 212 children and adolescents aged 0 to 18 years whose baseline (initial assessment) visits were between January 1, 2014, and December 31, 2018, and who were followed up until December 31, 2020, with at least 1 follow-up visit since the baseline visit. Patients with more than 6-month eligibility gaps during the study period were excluded from the analysis (details in eFigure 1 in Supplement 1). The EHR data contained information on patient demographic characteristics, medical history, health behavior, and clinical assessments for oral health conditions (diagnostic, caries risk assessment, and procedure codes). This study was reviewed by the institutional review board (IRB) of the Harvard Medical School and was determined to be exempt from the requirement of IRB approval and informed consent owing to the use of limited deidentified data. This study followed the Strengthening the Reporting of Observational Studies in Epidemiology (STROBE) reporting guidelines.

### Adherence to Quality Measures: TTE

**Figure 1.**
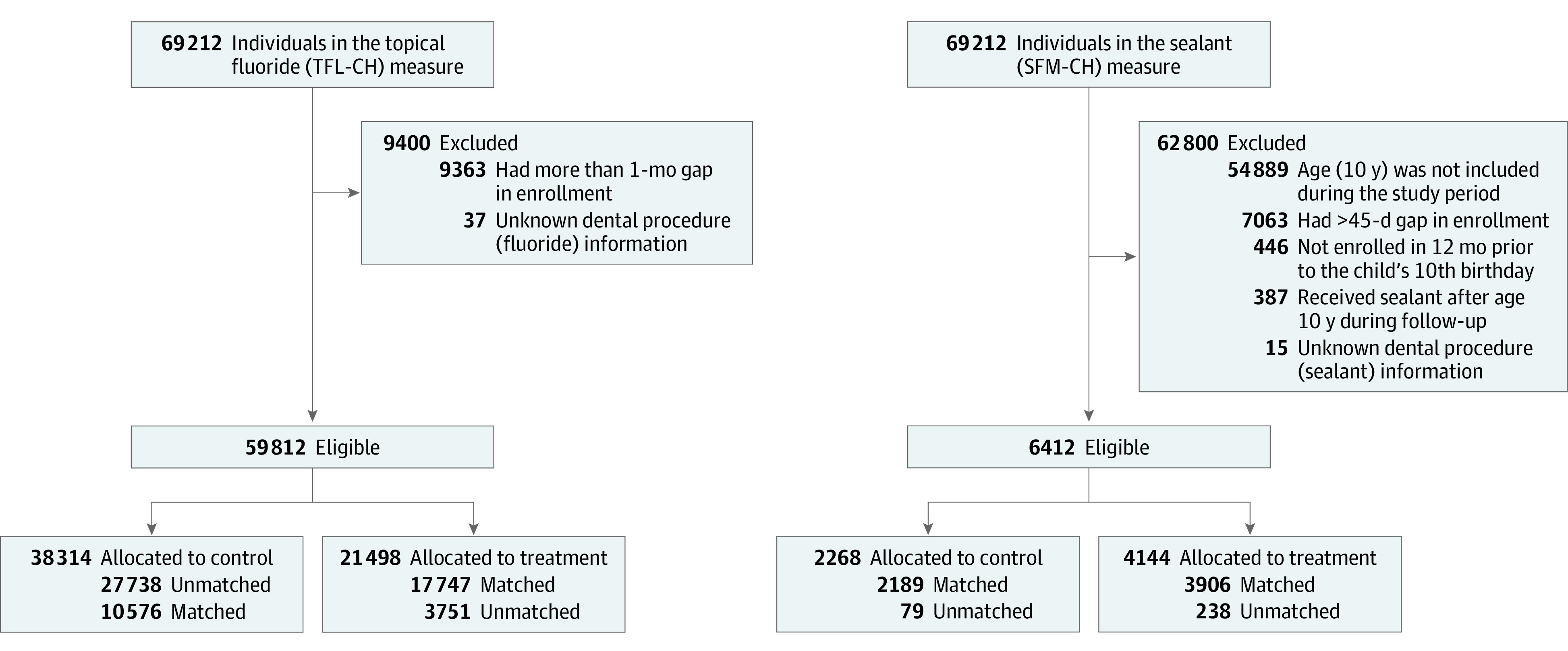
Flow Diagrams for Study Population for Target Trial Emulation Steps for identifying eligible individuals, determining a treatment strategy, and adjusting for baseline confounders to emulate random treatment assignment are shown.

Target trial emulation had a 2-step process.^19^ The first step was to fully articulate the causal question by specifying the protocol of the (hypothetical) trial; we examined how adhering to 2 pediatric Dental Quality Alliance quality measures included in the core set of children’s health care quality measures (receipt of ≥2 topical fluoride applications [TFL-CH] and placement of sealant on permanent first molars [SFM-CH]) is associated with the risk of developing tooth decay.^10,20^ The second step was to emulate the following components of the protocol using observational data: (1) identifying eligible individuals, (2) determining a treatment strategy, (3) following them up from assignment (time zero) until outcome or end of follow-up, and (4) adjusting for baseline confounders to emulate random treatment assignment (details in eMethods 1 in Supplement 1 and Figure 1). While comprehensive oral evaluation is included in the core set, we focused our analysis on receipt of fluoride and sealant because most of our cohort received oral evaluation, lacking enough control units to be matched and compared with treated units.

Eligibility criteria were determined based on the denominator condition (the given population to which a measure applies) for each quality measure (eMethods 1 in Supplement 1). Individuals who meet the numerator condition (the subset of patients in the denominator for whom a particular service has been provided) of the quality measures are considered adherent to quality measures (treated group). For each eligible individual, follow-up begins at the time of treatment assignment (whether they adhere to quality measures or not) and continues until the development of new tooth decay, loss to follow-up, or administrative end of follow-up (24 months after baseline). To emulate random treatment assignment conditional on baseline confounders (fourth component of the second step in TTE), coarsened exact matching was used to produce covariate balance between treated and control groups and to estimate average treatment effect with appropriate weights (eMethods 2 in Supplement 1). Coarsened exact matching categorizes original covariates into bins and then performs exact matching on subclasses formed by a complete cross of the coarsened covariates.^21^ Coarsened exact matching uses maximal information, resulting in strata that may include different numbers of treated and control units. To compensate for the differential strata sizes, weights are assigned for each unit. Subclasses that do not contain both treated and control units are discarded, leaving only subclasses containing treatment and control units that are exactly equal on the coarsened covariates. With this approach, the tradeoff between exact matching and distance matching can be managed to prevent discarding too many units.

### Measures

Our primary outcome was development of new tooth decay in either deciduous or permanent teeth, defined as at least 1 decayed, filled, or missing tooth due to caries, assessed by qualified dentists. Covariates included patients’ demographic characteristics (age at visit, gender, race and ethnicity, insurance type), smoking status, annual dental visit status (whether patients visited dental offices within a 12-month period), baseline caries risk assessment (low, moderate, or high [eTable 1 in Supplement 1]), dental procedures performed at baseline (binary indicators for oral evaluation, topical fluoride application, and sealant receipt [details available in eTable 1 in Supplement 1]), which were all found to be associated with the outcome.^22,23^ Insurance status was obtained at the baseline visit and categorized as commercially insured or public (Medicaid or Children’s Health Insurance Program). Gender was self-reported and classified as male, female, and unknown or transgender. Race and ethnicity of the patient were self-reported and classified as Asian, Black, Hispanic, White, other (including American Indian or Alaska Native and Native Hawaiian or Other Pacific Islander), multiracial, and unknown. Because unknown race and ethnicity information was associated with the likelihood of receiving recommended dental care, unknown race and ethnicity of individuals was categorized as missing to capture variation in dental care use across racial and ethnic groups (eTable 2 in Supplement 1).

### Sensitivity Analysis: Instrumental Variable Analysis

Because the possibility of unmeasured confounding cannot be excluded, we used near-far matching as a secondary analysis.^24^ Near-far matching is a 2-step approach to mimic a matched-pair RCT by matching individuals who are similar (near) on measured confounders and dissimilar (far) on an instrumental variable (IV). An IV is a factor that influences the outcome (tooth decay) only through its influence on the treatment variable (adherence to quality measures). Distance from residence to the clinic was our IV as it influences the likelihood of receiving recommended dental care but does not directly affect the risk of tooth decay. Near-far matching is used to strengthen IVs while maintaining the other benefits of matching.^24,25,26^ Our IV was found to be strong; via near-far matching, the *F* statistics (first stage) for predicting receipt of treatment increased from 31.32 to 38.14 for fluoride and from 9.94 to 14.67 for sealant. Additional details on IV and near-far matching approach are provided in eMethods 3 in Supplement 1.

### Statistical Analysis

Data were analyzed from May 1 to August 7, 2023. We first estimated Kaplan-Meier curves for the unadjusted cumulative incidence of tooth decay by treatment status (adherence to quality measures) for both prematched and weighted postmatched (on observed covariates) data. Then, we used an Andersen-Gill model, which generalizes the Cox proportional hazards regression model to account for time-varying covariates and thereby estimate the effect size of the adherence to quality measures adjusting for available covariates using the matched data (both matched on observed covariates and near-far matched data).^27^ In addition to the covariates included in the matching process, age at visit and receipt of other dental procedures during the follow-up period were included in the Andersen-Gill model and were allowed to vary over time.

Subgroup analyses were conducted to test for potential interaction with the adherence status using the matched (on observed covariates) data. The denominator (eligibility) criteria for the 2 quality measures (≥2 topical fluoride applications and sealant application) included children at elevated risk of caries until 2020. Although this condition was removed from the current measure specification, analyses were performed stratified by caries risk assessment status as low compared with elevated (moderate or high) (eTable 1 in Supplement 1). Given differences in payment and delivery systems between public and private insurance plans, subgroup analyses were conducted by insurance type (public [Medicaid or Children’s Health Insurance Program] vs commercially insured). Statistical significance was based on 2-sided *P* ≤ .05. All analyses were performed using R, version 3.6.1 (R Program for Statistical Computing).

## Results

### Study Cohort

**Figure 2.**
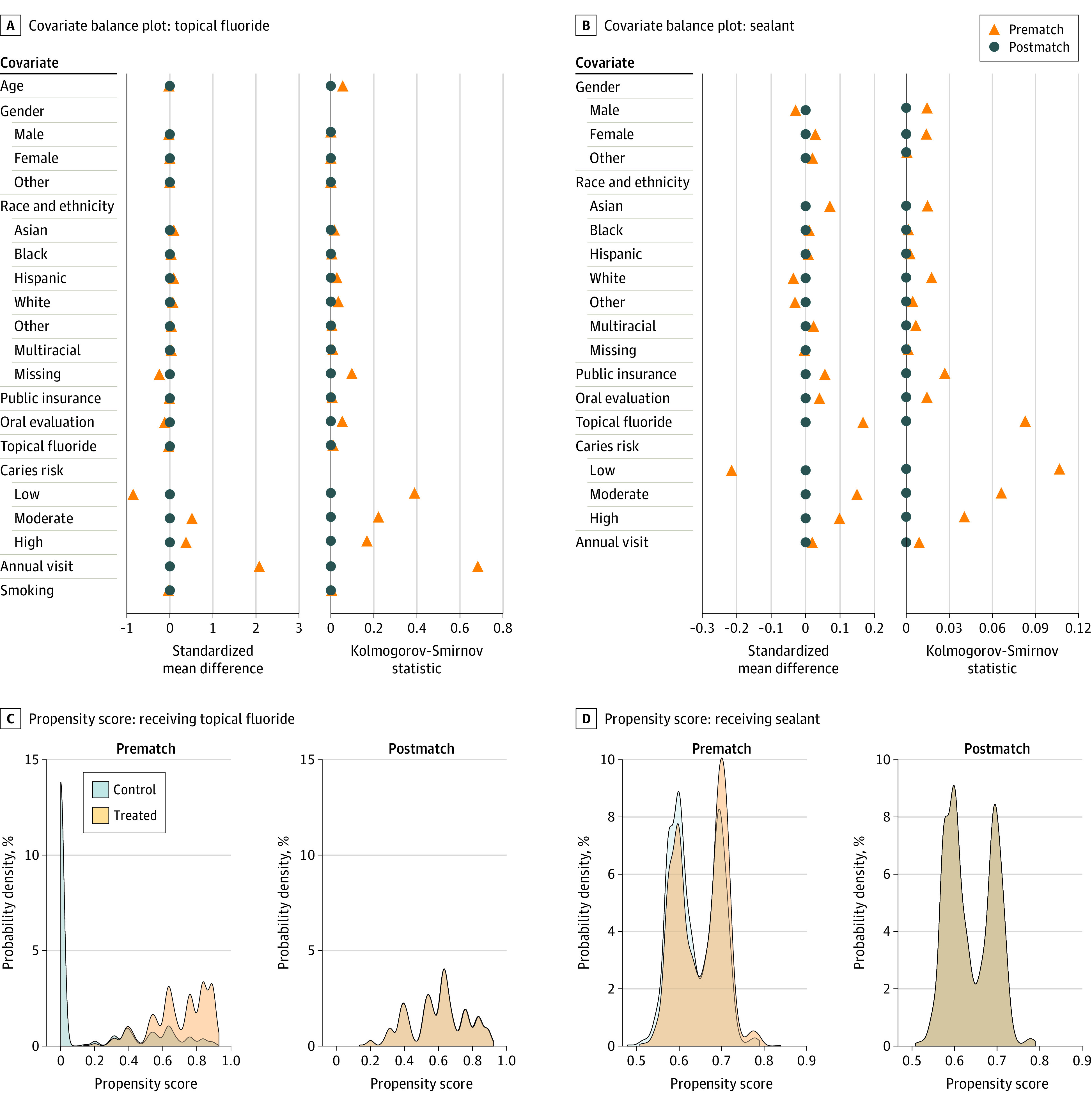
Balance Plots Before and After Matching Covariate balance plots and propensity score distributions are shown. Other gender indicates individuals with unknown or transgender identity. Other race and ethnicity includes American Indian or Alaska Native and Native Hawaiian or Other Pacific Islander.

Among 69 212 US children and adolescents aged 0 and 18 years (mean [SD] age, 10.2 [5.0] years), 34 241 (49.5%) were male, 34 915 (50.4%) were female, and 56 (0.1%) identified as unknown or transgender. In terms of race and ethnicity, 1930 (2.8%) participants were identified as Asian; 2038 (2.9%), Black; 8667 (12.5%), Hispanic; 33 632 (48.6%), White; and 22 945 (33.2%), other race or ethnicity, multiracial, or missing (eTable 2 in Supplement 1). Of 69 212 individuals in the total population sample (eFigure 1 in Supplement 1), 59 812 were eligible for receiving topical fluoride and 6412 were eligible for receiving sealant on permanent first molars (Figure 1). Among the 59 812 individuals eligible for fluoride, 21 498 (35.9%) received fluoride, and 17 747 (82.6%) of these were included in the matched cohort (Table 1 and eTable 2 in Supplement 1). In the matched cohort, median follow-up times were 11.9 (IQR, 6.6-25.2) months for the treated group and 8.8 (IQR, 3.6-24.9) months for the control group. For receipt of sealant, 4144 (64.6%) of 6412 eligible individuals were treated, and 3906 (94.3%) of these were included in the matched cohort. In the matched cohort, median follow-up times were 18.5 (IQR, 12.3-30.7) months for sealant recipients and 18.6 (IQR, 12.2-31.7) months for nonrecipients. Before matching, baseline characteristics between treated and control groups differed for both quality measures; while all 21 498 individuals who adhered to the fluoride measure had annual dental visits, only 13 741 (35.9%) of 38 314 in the control group had annual visits; for the sealant measure, 1623 (39.2%) in the treatment group and 871 (38.4%) in the control group had annual visits (Table 1). For both quality measures, treated groups had higher percentages of elevated (moderate or high) baseline caries risk than control groups (fluoride measure: 16 453 [76.5%] vs 15 236 [39.8%]; sealant measure: 2264 [54.6%] vs 997 [44.0%]). After matching, the absolute standardized mean differences and Kolmogorov-Smirnov statistics of baseline characteristics between adherent and nonadherent individuals were balanced (Figure 2). Propensity score distributions before and after matching were also compared between adherent and nonadherent individuals. Unmatched treated individuals were more likely to be at elevated risk of caries and publicly insured relative to matched treated individuals (eFigure 2 in Supplement 1). Following near-far matching, 27 504 (46.0%) of 59 812 eligible individuals were encouraged (those encouraged to receive treatment by living closer to the dental clinic, regardless of whether they adhered the quality measure) to receive fluoride, and 1855 (28.9%) of 6412 eligible individuals were encouraged to receive sealant. These encouraged individuals were matched to discouraged individuals. The near-far matched cohort was more balanced with absolute standardized differences less than 0.1 for all covariates (eTables 3 and 4 in Supplement 1).

**Table 1.** Study Population Characteristics Before and After Matching on Observed Covariates^a^

Characteristic	Study group for fluoride measure				Study group for sealant measure			
	Prematching (n = 59 812)		Postmatching (n = 28 323)^b^		Prematching (n = 6412)		Postmatching (n = 6095)^b^	
	Control (n = 38 314)	Treated (n = 21 498)	Control (n = 10 576)	Treated (n = 17 747)	Control (n = 2268)	Treated (n = 4144)	Control (n = 2189)	Treated (n = 3906)
Age, mean (SD), y	11.0 (5.2)	11.0 (5.0)	10.8 (4.8)	10.8 (4.5)	10.0 (NA)	10.0 (NA)	10.0 (NA)	10.0 (NA)
Age group, y								
<6	7292 (19.0)	3284 (15.3)	1718 (15.4)	2541 (14.9)	0	0	0	0
6-12	14 536 (37.9)	9963 (46.3)	4274 (45.5)	8673 (45.7)	2268 (100)	4144 (100)	2189 (100)	3906 (100)
13-18	16 486 (43.0)	8251 (38.4)	4584 (39.1)	6533 (39.4)	0	0	0	0
Gender								
Male	19 041 (49.7)	10 625 (49.4)	5138 (49.3)	8836 (49.3)	1140 (50.3)	2026 (48.9)	1106 (49.4)	1909 (49.4)
Female	19 243 (50.2)	10 852 (50.5)	5436 (50.7)	8910 (50.7)	1127 (49.7)	2114 (51.0)	1083 (50.5)	1997 (50.5)
Unknown or transgender	30 (0.1)	21 (0.1)	2 (0.01)	1 (0.01)	1 (0.04)	4 (0.1)	0	0
Race and ethnicity								
Asian	862 (2.2)	823 (3.8)	196 (2.0)	377 (2.1)	62 (2.7)	167 (4.0)	55 (2.7)	112 (2.7)
Black	913 (2.4)	630 (2.9)	175 (1.7)	318 (1.8)	57 (2.5)	111 (2.7)	40 (1.9)	75 (1.9)
Hispanic	3790 (9.9)	2746 (12.8)	976 (11.0)	2140 (12.1)	301 (13.3)	561 (13.5)	290 (13.2)	516 (13.2)
White	18 500 (48.3)	11 357 (52.8)	4962 (53.6)	10 211 (57.5)	1249 (55.1)	2211 (53.4)	1238 (56.2)	2186 (56.2)
Other^c^	717 (1.9)	522 (2.4)	172 (1.5)	246 (1.4)	56 (2.5)	82 (2.0)	43 (1.6)	52 (1.6)
Multiracial	3243 (8.5)	2072 (9.6)	831 (8.4)	1548 (8.7)	207 (9.1)	405 (9.8)	202 (9.6)	381 (9.5)
Missing	10 289 (26.9)	3348 (15.6)	3264 (21.8)	2907 (16.4)	336 (14.8)	607 (14.6)	321 (14.9)	584 (14.9)
Public insurance	14 867 (38.8)	7997 (37.2)	4252 (37.9)	6469 (37.9)	799 (35.2)	1572 (37.9)	760 (36.4)	1458 (36.4)
Smoking	641 (1.7)	247 (1.1)	94 (0.6)	87 (0.6)	0	0	0	0
Annual visit	13 741 (35.9)	21 498 (100)	10 576 (100)	17 747 (100)	871 (38.4)	1623 (39.2)	823 (37.7)	1473 (37.7)
Caries risk								
Low	23 078 (60.2)	5045 (23.5)	5123 (37.1)	4947 (34.8)	1271 (56.0)	1880 (45.4)	1244 (50.4)	1829 (50.4)
Moderate	6299 (16.4)	8185 (38.1)	1914 (27.9)	6265 (29.7)	548 (24.2)	1271 (30.7)	520 (28.0)	1188 (28.0)
High	8937 (23.3)	8268 (38.5)	3539 (35.0)	6535 (35.5)	449 (19.8)	993 (24.0)	425 (21.6)	889 (21.6)
Dental procedures^d^								
Evaluation	28 538 (74.5)	14 712 (68.4)	5916 (64.0)	12 538 (65.8)	1897 (83.6)	3530 (85.2)	1869 (86.5)	3402 (86.5)
Fluoride	13 623 (35.6)	17 197 (80.0)	2633 (30.4)	12 615 (67.1)	993 (43.8)	2161 (52.1)	974 (49.5)	2043 (49.5)
Sealant	6285 (16.4)	3088 (14.4)	1059 (10.1)	2085 (11.5)	0	903 (21.8)	0	865 (21.7)

Abbreviation: NA, not applicable.

^a^ The fluoride measure indicates receipt of topical fluoride at least twice; the sealant measure, placement of sealant on permanent teeth by 10 years of age. Unless otherwise indicated, data are expressed as No. (%) of individuals.

^b^ By default, coarsened exact matching (CEM) uses maximal information, resulting in strata that may include different numbers of treated and control units. To compensate for the differential strata sizes, weights are assigned for each unit. For postmatch data, percentages are weighted based on weights produced from CEM.

^c^ Includes American Indian or Alaska Native and Native Hawaiian or Other Pacific Islander.

^d^ Indicates percentages of individuals using each of these procedures at baseline.

### Adherence to Topical Fluoride Application and Oral Health Outcomes

**Figure 3.**
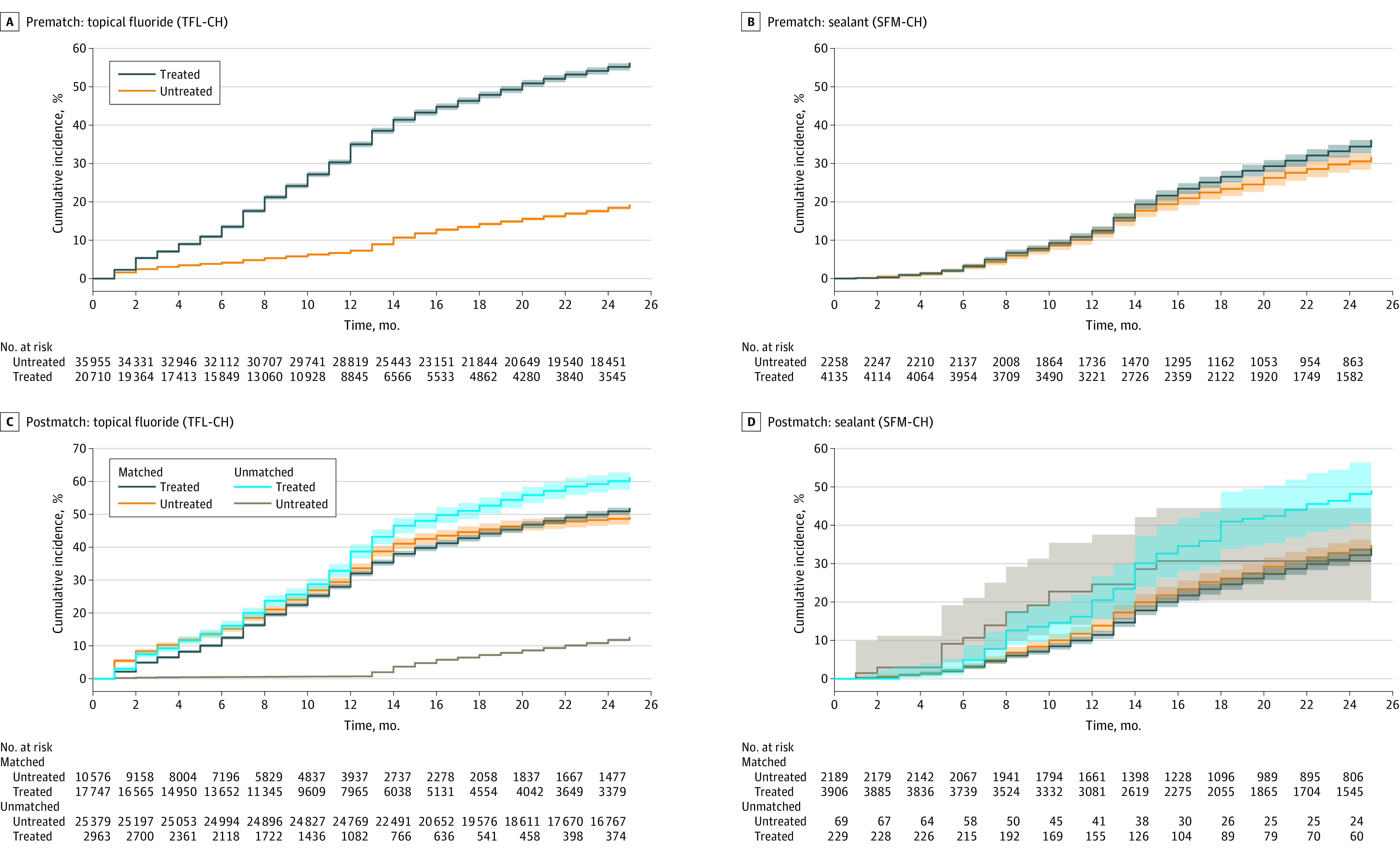
Cumulative Incidence of Tooth Decay Before and After Matching Kaplan-Meier curves are unadjusted and estimated for time to first tooth decay event on the raw data (before matching) and target trial emulation matched data (after matching) without adjusting for time-varying covariates during the follow-up period. For data after matching, matching status (matched vs unmatched) was incorporated in estimating the curve.

During a 24-month follow-up, 47.9% (95% CI, 46.9%-49.0%) of participants experienced tooth decay in the matched cohort for fluoride treatment. Those receiving fluoride were at a lower risk of tooth decay compared with individuals who did not (unadjusted hazard ratio [HR], 0.91 [95% CI, 0.86-0.96]) (Table 2). The result differed from the prematched data: 30.5% (95% CI, 30.1%-31.0%) experienced tooth decay with an HR of 4.05 (95% CI, 3.91-4.19) for those receiving fluoride compared with the control group (Figure 3). When all available and time-varying covariates during the follow-up period were adjusted, adhering to the fluoride measure was associated with reduced risk of tooth decay with an adjusted HR (AHR) of 0.82 (95% CI, 0.78-0.86) in the matched cohort (Table 2 and eFigure 3 in Supplement 1). In the near-far matched cohort, the AHR was 0.84 (94% CI, 0.76-0.92). Adhering to fluoride treatment was associated with greater risk reduction among children at elevated risk and with public insurance, with AHRs of 0.80 (95% CI, 0.76-0.85) and 0.72 (95% CI, 0.67-0.78), respectively.

**Table 2.** Incidence of Tooth Decay Between Adherent and Nonadherent Individuals

Group	No. of participants	HR (95% CI)	
		Unadjusted^a^	Adjusted^b^
**Receiving topical fluoride at least twice**			
Overall			
Matched on treatment status^c^			
Control	10 576	1 [Reference]	1 [Reference]
Treated	17 747	0.91 (0.86-0.96)	0.82 (0.78-0.86)
Near-far matched on IV^d^			
Control	34 708	1 [Reference]	1 [Reference]
Treated	20 300	4.00 (3.73-4.30)	0.84 (0.76-0.92)
Subgroup			
Elevated baseline risk			
Control	5591	1 [Reference]	1 [Reference]
Treated	13 150	0.84 (0.79-0.89)	0.80 (0.76-0.85)
Public insurance			
Control	4252	1 [Reference]	1 [Reference]
Treated	6469	0.77 (0.71-0.83)	0.72 (0.67-0.78)
**Receiving sealant by 10 y of age**			
Overall			
Matched on treatment status^c^			
Control	2189	1 [Reference]	1 [Reference]
Treated	3906	0.94 (0.83-1.05)	0.86 (0.76-0.97)
Near-far matched on IV^d^			
Control	1396	1 [Reference]	1 [Reference]
Treated	2314	1.17 (1.02-1.33)	0.87 (0.75-0.99)
Subgroup			
Elevated baseline risk			
Control	945	1 [Reference]	1 [Reference]
Treated	2077	0.87 (0.76-0.99)	0.83 (0.72-0.95)
Public insurance			
Control	760	1 [Reference]	1 [Reference]
Treated	1458	0.84 (0.69-1.01)	0.78 (0.65-0.94)

Abbreviation: IV, instrumental variable.

^a^ Only incorporates matching.

^b^ Incorporates other time-varying covariates in addition to matching at baseline.

^c^ Matched on observed covariates between adherent and nonadherent individuals.

^d^ Matches individuals similar (near) on observed covariates and dissimilar (far) on values of an IV.

### Adherence to Sealant Receipts and Oral Health Outcomes

In the prematched data, 31.7% (95% CI, 29.5%-34.1%) of participants experienced tooth decay during a 24-month follow-up, and treated individuals appeared to experience a higher rate of tooth decay with an unadjusted HR of 1.15 (95% CI, 1.04-1.27) (Figure 3). In the matched cohort, 31.5% (95% CI, 29.3%-34.0%) experienced tooth decay during a 24-month follow-up, and the risk of experiencing tooth decay was not lower in the treated group relative to the control group when unadjusted for other covariates (HR, 0.94 [95% CI, 0.83-1.05]) (Table 2). When fully adjusted, receipt of sealant was associated with reduced risk of tooth decay with an AHR of 0.86 (95% CI, 0.76-0.97) in the matched cohort (Table 2 and eFigure 3 in Supplement 1). In the near-far matched cohort, the AHR was 0.87 (95% CI, 0.76-0.99). Among children at elevated risk and with public insurance, receipt of sealant was associated with a greater reduction in the risk of tooth decay, with AHRs of 0.83 (95% CI, 0.72-0.95) and 0.78 (95% CI, 0.65-0.94), respectively (Table 2).

## Discussion

Our retrospective cohort study used a TTE framework to estimate the association between adhering to 2 pediatric dental quality measures and the risk of developing tooth decay among US children by emulating hypothetical RCTs and found that adhering to topical fluoride and sealant measures was associated with reduced risk of tooth decay. Benefits of adhering to these quality measures were greater among children at elevated risk and those with public insurance compared with those at low risk and with commercial insurance. Under most payment policies currently in place in the US, dental clinicians would be incentivized to complete procedures irrespective of treatment outcomes. Our study allows us to better understand the link between adherence to dental quality measures and health outcomes and illustrates the needs for improvement in quality assessment in dentistry.

Randomized clinical trials are the criterion standard for studying causal relationships by ensuring that treatment and control groups are comparable, and thus any differences in the outcome can be attributed to the treatment or intervention. Clearly specified start of follow-up for each individual is another reason that RCTs can provide unbiased and consistent estimates of causal effects.^19^ Several RCTs and meta-analysis studies have evaluated the effectiveness of topical fluoride and sealant in preventing dental caries among children^13,14,15^; however, heterogeneities among studies were observed.^17,18^ Many of these RCTs were school-based programs, often targeting school-aged children in low socioeconomic status neighborhoods.^28^ Eligibility criteria for some of these RCTs varied among studies and were different from the eligibility criteria (denominator condition) specified by dental quality measures. For these reasons, it is challenging to identify associations between adherence to dental quality measures and oral health outcomes that can be generalizable to other populations. Our study demonstrates that TTE methods using observational data can be used to estimate average treatment effects of adhering to process-based quality measures on time to tooth decay events by emulating target trials that can be extended to broader populations to whom these quality measures are applicable, complementing evidence from RCTs.

While value-based care (VBC) models have gained considerable attention in medicine, oral health care has faced challenges in implementing a VBC system, influenced by multiple factors. One of the biggest challenges is that developed process measures lack input from diagnostic or clinical data sources that link to oral health outcomes; current dental quality measures are designed to evaluate program operations rather than population health. A VBC model in dentistry needs simultaneously to incentivize increased access to care and the measurement of outcomes, ultimately tying payment to those outcomes.^29,30^ By quantifying the link between adherence to quality measures and patient health outcomes, our study provides insights in the process-outcome association and prioritizing evidence-based quality measures. Additionally, quality measures and incentives must acknowledge the presence of oral health disparities and should not disincentivize clinicians from providing care to at-risk populations. Our study found that certain high-risk subpopulations, such as children with public insurance, may experience greater benefits from measure adherence on health outcomes. On average, Medicaid reimbursement rates for child dental services are approximately 61.4% of private insurance reimbursement,^31^ potentially disincentivizing clinicians from providing care. Identification of vulnerable subgroups at high risk for poor outcomes has the potential to inform oral health policy interventions, such as Medicaid reimbursement policies, and to facilitate more tailored quality improvement efforts, such as quality reporting stratified by population characteristics.^32^

### Limitation

Our study has limitations. First, we assumed no unmeasured confounding (strong ignorability assumption). Although observational cohorts are created via matching to be exchangeable across a set of measure covariates, we cannot prove that there is no omitted variable bias.^33^ Clinical data sources, including EHR data, often lack certain types of information, such as social determinants of health (eg, poverty and educational attainment) that are known to influence health care decisions and health outcomes.^34^ Therefore, lack of such information could confound an observational study. In our study, we performed near-far matching (distance to clinics as an IV) as a sensitivity analysis to adjust for unmeasured confounding and observed that the effect estimates were close to the estimates obtained from our matched analyses. Additionally, because our study sample was a convenience sample of children who were enrolled and received care at large dental accountable care organizations across 3 states, the findings may not be generalizable beyond these populations.

## Conclusions

In this cohort study, receipt of topical fluoride and sealant among children and adolescents eligible for these treatments was found to be associated with a reduced risk of tooth decay, and the benefits were greater among vulnerable subgroups of population. These findings provide insights in facilitating targeted application of quality measures or developing more tailored quality measures. Supporting application of fluoride and sealant in nondental settings and by primary care clinicians would have potential to increase access to recommended dental care and thus, improve children’s oral health.

## Notes

Supplement 1. **eMethods 1.** Target Trial Emulation and Dental Quality Measures **eMethods 2.** Coarsened Exact Matching **eMethods 3.** Near-Far Matching **eTable 1.** Dental Procedure Codes Used for Procedure Grouping and Baseline Caries Risk **eTable 2.** Baseline Characteristics of the Total and Eligible Populations for Quality Measures **eTable 3.** Baseline Characteristics Before and After Near-Far Matching for Topical Fluoride **eTable 4.** Baseline Characteristics Before and After Near-Far Matching for Sealant **eFigure 1.** CONSORT Flow Diagram for Study Population and Target Trial Emulation **eFigure 2.** Flow Diagrams for Target Trial Emulation and Patient Characteristics in Matched and Unmatched Groups **eFigure 3.** Results of Time-to-Event Regression Models
eReferences

Supplement 2.
Data Sharing Statement
