# Supplementary material for: Quality Measure Adherence and Oral Health Outcomes in Children
Source: JAMA Netw Open. 2024 Jan 30;7(1):e2353861. doi: 10.1001/jamanetworkopen.2023.53861 (PMC10828912; doi:10.1001/jamanetworkopen.2023.53861)
Supplement: Supplement 1. — eMethods 1. Target Trial Emulation and Dental Quality Measures eMethods 2. Coarsened Exact Matching eMethods 3. Near-Far Matching eTable 1. Dental Procedure Codes Used for Procedure Grouping and Baseline Caries Risk eTable 2. Baseline Characteristics of the Total and Eligible Populations for Quality Measures eTable 3. Baseline Characteristics Before and After Near-Far Matching for Topical Fluoride eTable 4. Baseline Characteristics Before and After Near-Far Matching for Sealant eFigure 1. CONSORT Flow Diagram for Study Population and Target Trial Emulation eFigure 2. Flow Diagrams for Target Trial Emulation and Patient Characteristics in Matched and Unmatched Groups eFigure 3. Results of Time-to-Event Regression Models eReferences [file jamanetwopen-e2353861-s001.pdf]

Supplementary Online Content

Choi SE, Pandya A, White J, Mertz E, Normand S-L. Quality measure adherence and oral health outcomes in children. *JAMA Netw Open*. 2024;7(1):e2353861. doi:10.1001/jamanetworkopen.2023.53861

eMethods 1. Target Trial Emulation and Dental Quality Measures
eMethods 2. Coarsened Exact Matching
eMethods 3. Near-Far Matching
eTable 1. Dental Procedure Codes Used for Procedure Grouping and Baseline Caries Risk
eTable 2. Baseline Characteristics of the Total and Eligible Populations for Quality Measures
eTable 3. Baseline Characteristics Before and After Near-Far Matching for Topical Fluoride
eTable 4. Baseline Characteristics Before and After Near-Far Matching for Sealant
eFigure 1. CONSORT Flow Diagram for Study Population and Target Trial Emulation
eFigure 2. Flow Diagrams for Target Trial Emulation and Patient Characteristics in Matched and Unmatched Groups
eFigure 3. Results of Time-to-Event Regression Models
eReferences.

This supplementary material has been provided by the authors to give readers additional information about their work.

© 2024 Choi SE et al. *JAMA Network Open*.

eMethods 1. Target Trial Emulation and Dental Quality Measures

When quantifying the effect of a treatment on a clinical outcome between a group of patients who administer treatment and a group who do not, one of the key advantages of a randomized trial is that both groups are expected to be comparable, and thus any differences in health outcomes at the end of the trial can be attributed to treatment rather than to prognostic differences between the groups. In addition, the start of follow-up for each participant is clearly specified (time of randomization) in a randomized trial. These are important features that are difficult to be achieved when drawing causal conclusions from observational studies.

A target trial ensures that observational studies preserve these features of randomized trials, and thus, emulate a hypothetical randomized trial.[1] Target trial emulation is a 2-step process as listed below.[2]

a. The first step is articulating the causal question in the form of the protocol of a hypothetical randomized trial that would provide the answer.
b. The second step is explicitly emulating the components of that protocol using the observational data: 1) finding eligible individuals, 2) assigning them to a treatment strategy compatible with their data, 3) following them up from assignment (time zero) until outcome or end of follow-up, and 4) conducting the same analysis as the corresponding target trial, except that there is adjustment for baseline confounders in an attempt to emulate random treatment assignment.

Following the target trial emulation framework,

a. We sought to evaluate the impact of adhering to two of Dental Quality Alliance (DQA) quality measures on the risk of developing new tooth decay: Receipt of at least two topical fluoride applications (TFL-CH) and sealant on permanent first molars (SFM-CH).[3]

b. Based on the technical specification for two quality measures below, 1) eligible individuals will be determined based on age, denominator, and enrollment criteria (CONSORT diagram in Supplemental eFigure 1) ; 2) treatment assignment will be determined based on numerator; 3) time zero will be at a time point when each individual meets the numerator condition; 4) treated (adherent to quality measures) and untreated (non-adherent to quality measures) were matched on baseline confounders.

	Sealant Receipt on Permanent First Molars (SFM-CH)	Topical Fluoride for Children (TFL-CH)
Description	Percentage of enrolled children who have ever received sealants on permanent first molar teeth. Two rates are reported: (1) at least one sealant and (2) all four molars sealed by the 10th birthdate.	Percentage of enrolled children ages 1 through 20 who received at least two topical fluoride applications as: (1) dental or oral health services, (2) dental services,

© 2024 Choi SE et al. *JAMA Network Open*.

		and (3) oral health services within the measurement year
Age	Children who turn age 10 in the measurement year.	Children ages 1 through 20
Denominator	Children who turn age 10 in the measurement year.	Children ages 1 through 20 as of the measurement year
Continuous enrollment	12 months prior to the child's 10th birthdate	The measurement year
Allowable gap in enrollment	No more than one gap in enrollment of up to 45 days during the continuous enrollment period.	No more than one gap in enrollment of up to 31 days during the measurement year.
Numerator	The unduplicated number of enrolled children who ever received a sealant on:<br>• At least one permanent first molar tooth<br>• All four permanent first molars.	The unduplicated number of enrolled children who received at least two fluoride applications as the following during the measurement year: • Dental or oral health services<br>• Dental services<br>• Oral health services Fluoride applications must be provided on at least two unique dates of service.

© 2024 Choi SE et al. *JAMA Network Open*.

eMethods 2. Coarsened Exact Matching

Matching is a nonparametric method of preprocessing data to control for some or all of the potentially confounding influence of pretreatment control variables by reducing imbalance between the treated and control groups. Coarsened exact matching (CEM) is a monotonic imbalance bounding method that categorizes original covariates into bins and then performs exact matching on subclasses formed by a complete cross of the coarsened covariates.[4] By default, CEM uses maximal information, resulting in strata that may include different numbers of treated and control units. To compensate for the differential strata sizes, weights are assigned for each unit. Subclasses that do not contain both treated and control units are discarded, leaving only subclasses containing treatment and control units that are exactly equal on the coarsened covariates. With CEM, tradeoff between exact matching and distance matching can be managed to prevent discarding too many units.

There are two weights in each subclass; one for the treated units and the other for control units. In each subclass, weights are calculated to maintain the overall proportion of treated and control groups within each subclass after weighting.

$$\frac{N_t}{N_c} = \frac{w_t n_t}{w_c n_c}$$

$$n_t + n_c = w_t n_t + w_c n_c$$

where:
w: Weight for a record
n: Number of matched records in the subclass
N: Number of total matched records
t for treated and c for control

To obtain weights for average treatment effect (ATE), both treated and control groups are weighted as follows,

Weights for treated group: $w_t = \frac{n_t + n_c}{n_t\,(1 + \frac{N_c}{N_t})}$

Weights for control group: $w_c = \frac{n_t + n_c}{n_c\,(1 + \frac{N_t}{N_c})}$

© 2024 Choi SE et al. *JAMA Network Open*.

eMethods 3. Near-Far Matching

Instrumental variable (IV) analysis is a method widely used to account for unmeasured confounding.[5] The IV is a variable associated with the treatment assignment, it affects the outcome only through the exposure and it is independent of confounders. In our study, we chose "distance from residence to dental clinic" as an IV because it influences the likelihood of receiving recommended dental care but does not directly affect the risk of tooth decay (below figure shows the relationship between distance to clinic and probability of adhering to quality measures).

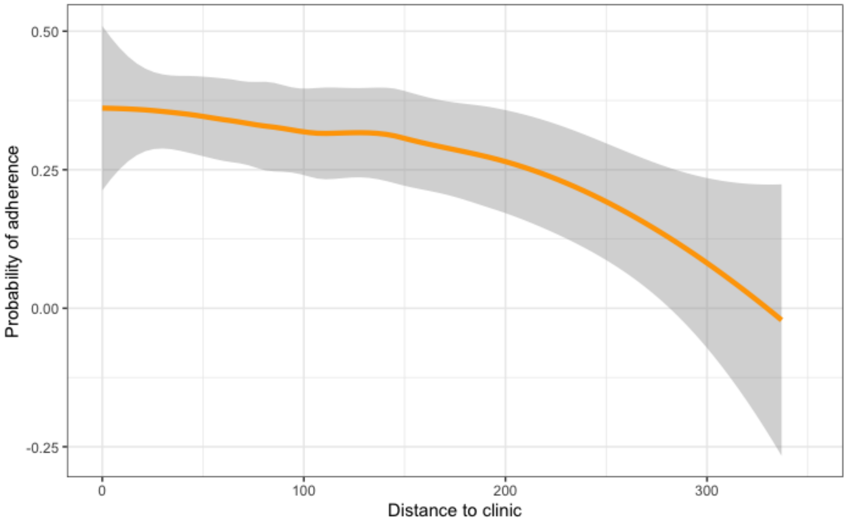

The function of distance as an instrument in this study was justified on the basis of 1) being likely to affect adherence to quality measures, 2) not being associated with the outcome except by receipt of the treatment, and 3) there being no expected confounding between the IV and the treatment. We formally test the first assumption by examining the strength of the instrument using the F-statistic for a regression of distance on receipt of treatment and by examining how the odds of treatment decreased as one lived farther away from the clinic. The second and third assumptions are not formally testable, as there could always be unmeasured confounding. Instead, to support the role of distance as an instrument in this case, we compared balance tables categorized by distance and by treatment status and observed that balance on important confounding variables improves, even without matching (Table S3 and Figure 2). This suggests that there is an element of randomization introduced by the IV for the observed covariates.

The first stage F-statistic for predicting the receipt of treatment was by the IV was 31.32 for foride and 9.94 for sealant. and the odds ratios for receipt by the IV were 0.97 (95% CI: 0.96-0.98) for floride and 0.99 (95% CI: 0.98-1.00) for sealant.

To adjust for covariate imbalance between those who adhere to quality measures and those who do not and strength the weak IV, we conducted near-far matching, a newer form of instrumental variable analysis that can improve the strength of an instrument

© 2024 Choi SE et al. *JAMA Network Open*.

and further reduce bias.[6,7] Near-far matching mimics a matched-pair randomized trial, generating weights to match individuals to be as similar as possible (“near”) in their observed characteristics, but as different as possible (“far”) in their value of the instrumental variable. Near/far matching helps augment the influence of an instrument; the instrument will help make an individual more likely to adhere to quality measures than its counterpart that is otherwise similar. We performed near-far instrumental variable analysis by 1:1 matching pairs of individuals that are as similar as possible among all covariates listed in Supplemental Table S3, while simultaneously being as different as possible in values of the instrumental variable. Near-far matching can strengthen an instrumental variable and reduce bias in the effect size estimates,[7,8] while providing benefits of matching on observed covariates—namely, reduced model dependency, nonparametric adjustment for measured confounders, and lowered mean squared error in the estimated effects of the predictor variable.[9] By selecting a matched sample and applying an instrumental variable analysis, however, the near-far approach privileges strengthening inference in terms of reducing bias and mean squared error, while reducing generalizability in the assessment by removing unmatched counties from the national sample.

Near/far matches are controlled by two key parameters: the percent sinks (the percentage of sample to be lost as unsuitable matches due to inadequate common support for inference), and the cut-point of differentiation for the instrumental variable that specifies the difference in instrument values in the pair match below which strong penalties are enforced. Simulated annealing is used to find the percent sinks and cut-point maximizing the partial F statistic. To get the closest covariate balance between the two groups, and at the same time achieve maximal separation in the values of the instrumental variable, some individuals are not matched.

For topical fluoride application measure, the near/far matching process increased the partial F statistics from 31.32 to 38.14 (the optimal percent sinks to 8.17%), reducing the dataset to a total of 23,009 matched pairs of individuals. For sealant measure, the near/far matching increased the partial F statistics from 9.94 to 14.67 (the optimal percent sinks to 15.08%), filtering the cohort to a total of 2405 matched pairs. The matched cohort was more balanced with absolute standardized differences less than 0.1 for all covariates (Supplemental Table S3).

© 2024 Choi SE et al. *JAMA Network Open*.

eFigure 1. CONSORT Flow Diagram for Study Population and Target Trial Emulation

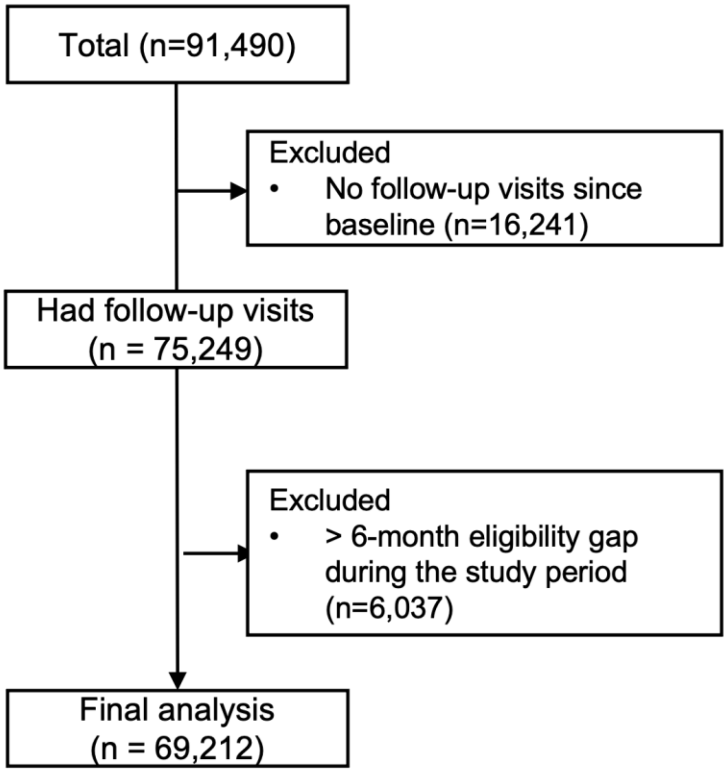

© 2024 Choi SE et al. *JAMA Network Open*.

eFigure 2. Flow Diagrams for Target Trial Emulation and Patient Characteristics in Matched and Unmatched Groups

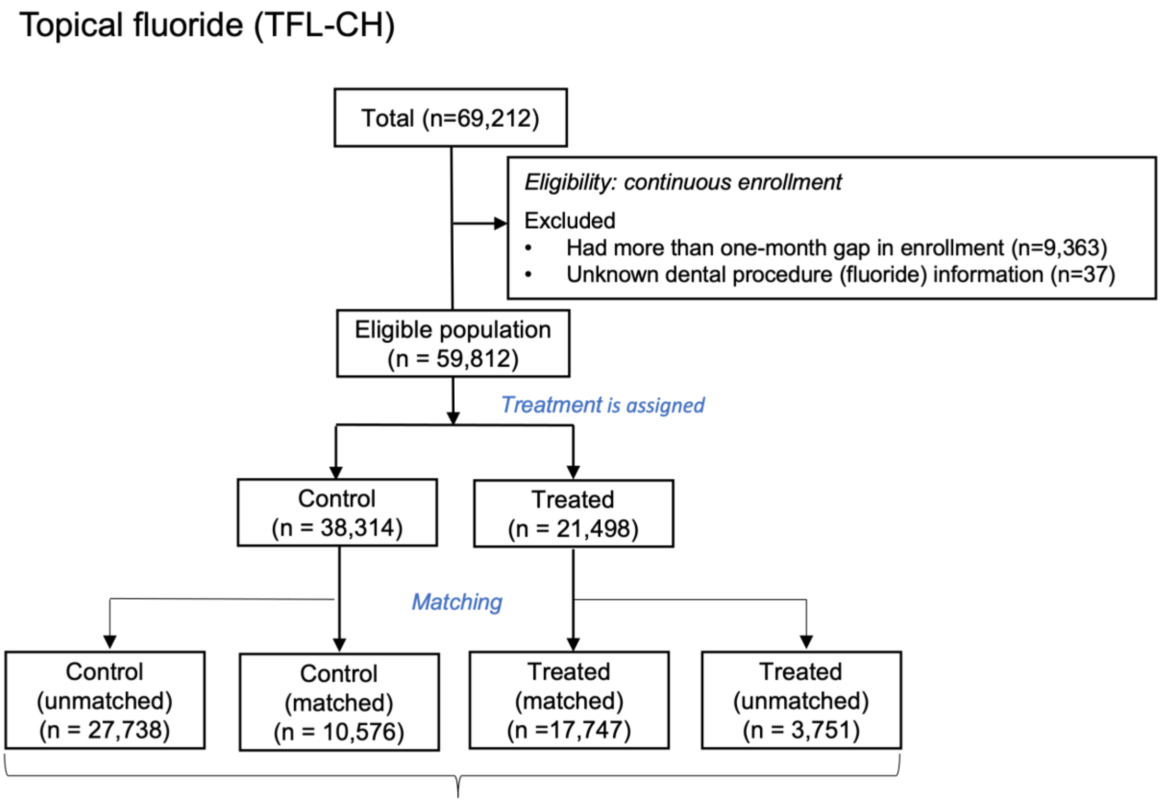

	Control (n=38,314)		Treated (n=21,498)	
	Unmatched (n=27,738)	Matched (n=10,576)	Unmatched (n=3,751)	Matched (n=17,747)
Age at baseline	11.11 (5.31)	10.77 (4.83)	13.80 (6.23)	10.83 (4.54)
Sex				
Male	13903 (50.1)	5138 (49.3)	1789 (47.7)	8836 (49.3)
Female	13807 (49.8)	5436 (50.7)	1942 (51.8)	8910 (50.7)
GenderX	28 (0.1)	2 (0.0)	20 (0.5)	1 (0.0)
Race				
Asian	666 (2.4)	196 (2.0)	446 (11.9)	377 (2.1)
Black	738 (2.7)	175 (1.7)	312 (8.3)	318 (1.8)
Latino	2814 (10.1)	976 (11.0)	606 (16.2)	2140 (12.1)
White	13538 (48.8)	4962 (53.6)	1146 (30.6)	10211 (57.5)
Other	545 (2.0)	172 (1.5)	276 (7.4)	246 (1.4)
Multiracial	2412 (8.7)	831 (8.4)	524 (14.0)	1548 (8.7)
Missing	7025 (25.3)	3264 (21.8)	441 (11.8)	2907 (16.4)
Public insurance	10615 (38.3)	4252 (37.9)	1528 (40.7)	6469 (37.9)
Smoking	550 (2.0)	94 (0.6)	164 (4.4)	87 (0.6)
Annual visit	3165 (11.4)	10576 (100.0)	3751 (100.0)	17747 (100.0)
Baseline caries risk				
Low	18093 (65.2)	5123 (37.1)	448 (11.9)	4947 (34.8)
Moderate	4461 (16.1)	1914 (27.9)	1778 (47.4)	6265 (29.7)
High	5184 (18.7)	3539 (35.0)	1525 (40.7)	6535 (35.5)
Dental procedures				
Evaluation	23081 (83.2)	5916 (64.0)	2227 (59.4)	12538 (65.8)
Fluoride	11124 (40.1)	2633 (30.4)	2583 (68.9)	12615 (67.1)
Sealant	5114 (18.4)	1059 (10.1)	989 (26.4)	2085 (11.5)

© 2024 Choi SE et al. *JAMA Network Open*.

Sealant (SFM-CH)

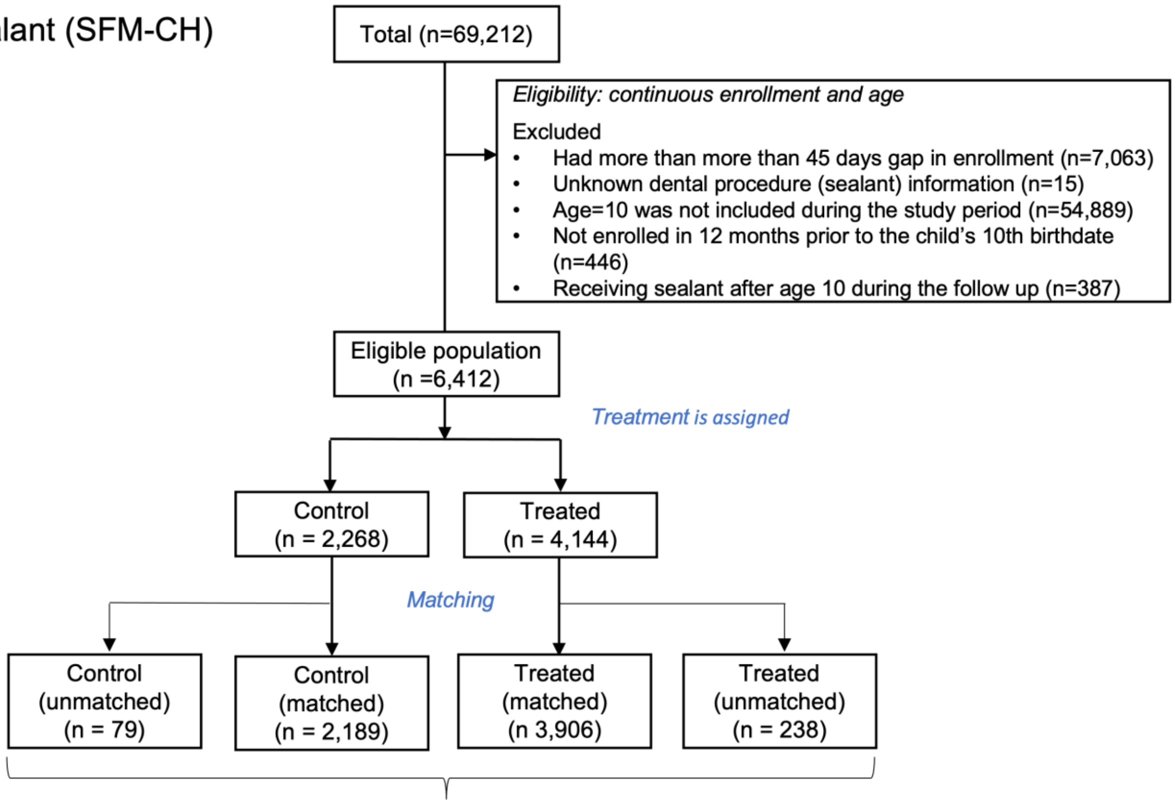

	Control (n=2,268)		Treated (n=4,144)	
	Unmatched (n=79)	Matched (n=2,189)	Unmatched (n=238)	Matched (n=3,906)
Age at baseline	10.00 (0.00)	10.00 (0.00)	10.00 (0.00)	10.00 (0.00)
Sex				
Male	34 (43.0)	1106(49.4)	117 (49.2)	1909 (49.4)
Female	44 (55.7)	1083(50.5)	117 (49.2)	1997 (50.5)
Gender X	1 (1.3)	0 (0.0)	4 (1.7)	0 (0.0)
Race				
Asian	7 (8.9)	55 (2.7)	55 (23.1)	112 (2.7)
Black	17 (21.5)	40 (1.9)	36 (15.1)	75 (1.9)
Hispanic	11 (13.9)	290 (13.2)	45 (18.9)	516 (13.2)
White	11 (13.9)	1238(56.2)	25 (10.5)	2186(56.2)
Other	13 (16.5)	43 (1.6)	30 (12.6)	52 (1.6)
Multiracial	5 (6.3)	202 (9.6)	24 (10.1)	381 (9.5)
Missing	15 (19.0)	321 (14.9)	23 (9.7)	584 (14.9)
Public insurance	39 (49.4)	760 (36.4)	114 (47.9)	1458(36.4)
Smoking	79 (100.0)	0 (0.0)	238 (100.0)	0 (0.0)
Annual visit	48 (60.8)	823 (37.7)	150 (63.0)	1473 (37.7)
Baseline caries risk				
Low	27 (34.2)	1244(50.4)	51 (21.4)	1829 (50.4)
Moderate	28 (35.4)	520 (28.0)	83 (34.9)	1188 (28.0)
High	24 (30.4)	425 (21.6)	104 (43.7)	889 (21.6)
Dental procedures				
Evaluation	28 (35.4)	1869(86.5)	128 (53.8)	3402 (86.5)
Fluoride	19 (24.1)	974 (49.5)	118 (49.6)	2043 (49.5)
Sealant	0 (0.0)	0 (0.0)	38 (16.0)	865 (21.7)

© 2024 Choi SE et al. *JAMA Network Open*.

eFigure 3. Results of Time-to-Event Regression Models

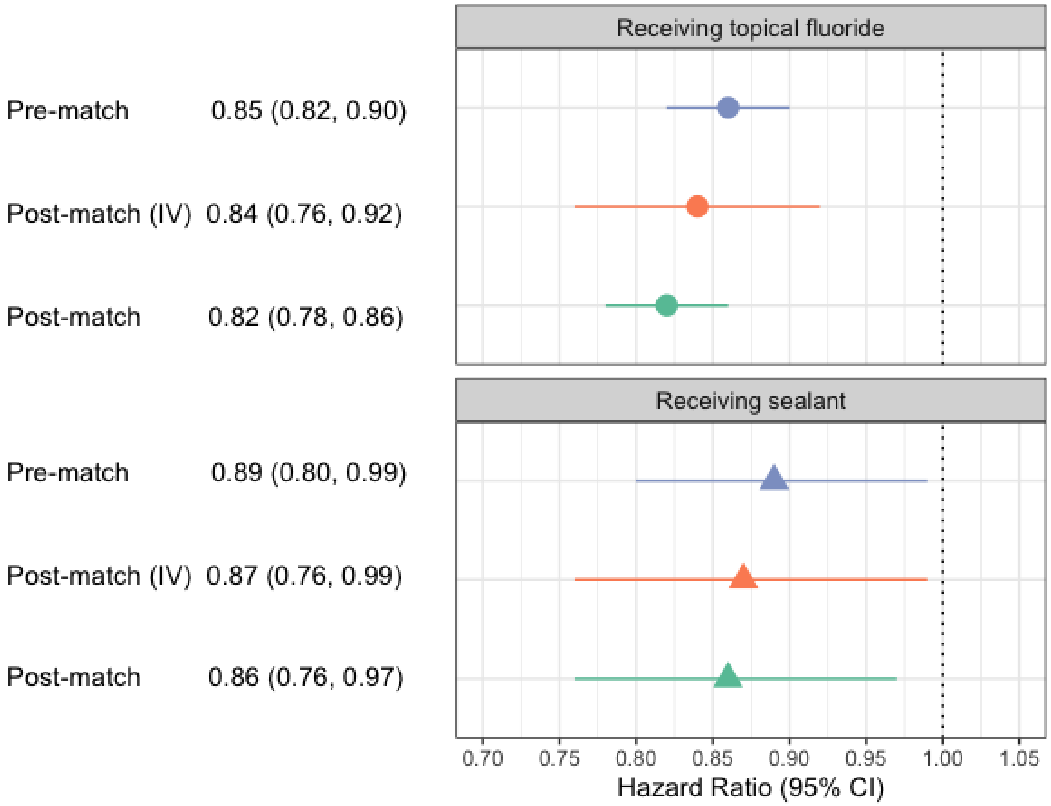

Note: Estimates are obtained from estimating Anderson Gill models using raw data before matching ("pre-match"), target trial emulation matched data ("post-match"), and near/far matched data ["post-match (IV)"], adjusting for time-varying covariates. Error bars indicate 95% confidence intervals around the hazard ratio estimates.

© 2024 Choi SE et al. *JAMA Network Open*.

eTable 1. Dental Procedure Codes Used for Procedure Grouping and Baseline Caries Risk

**Grouping**		**CDT procedure codes**
Cleaning		D0120, D0140, D0145, D0150, D0170
Fluoride application		D1206, D1208
Sealant		D1351, D1353
Caries risk	Low	D0601
	Moderate	D0602
	High	D0603

Caries risk assessment is part of a comprehensive treatment plan approach, estimating children at low, moderate, and high risk of caries based on a child's age, social/behavioral/medical risk factors (i.e., low health literacy, high sugar consumption, special health care needs), protective factors (i.e., tooth brushing frequency, exposure to fluoride) and clinical findings (i.e. visible plaque on teeth, presence of enamel defects).[10]

© 2024 Choi SE et al. *JAMA Network Open*.

eTable 2. Baseline Characteristics of the Total and Eligible Populations for Quality Measures

	**Overall** (n=69,212)	**Eligible for topical fluoride** (n=59,812)		**Eligible for sealant** (n=6,412)	
		Control (n=38,314)	Treated (n=21,498)	Control (n=2,268)	Treated (n=4,144)
Age at baseline	10.23 (5.03)	11.02 (5.16)	10.95 (5.02)	10.00 (0.00)	10.00 (0.00)
Age group					
< 6	15336 (22.2)	7292 (19.0)	3284 (15.3)		
6-12	28807 (41.6)	14536 (37.9)	9963 (46.3)	2268 (100.0)	4144 (100.0)
13-18	25069 (36.2)	16486 (43.0)	8251 (38.4)		
Sex					
Male	34241 (49.5)	19041 (49.7)	10625 (49.4)	1140 (50.3)	2026 (48.9)
Female	34915 (50.4)	19243 (50.2)	10852 (50.5)	1127 (49.7)	2114 (51.0)
Gender X	56 (0.1)	30 (0.1)	21 (0.1)	1 (0.0)	4 (0.1)
Race					
Asian	1930 (2.8)	862 (2.2)	823 (3.8)	62 (2.7)	167 (4.0)
Black	2038 (2.9)	913 (2.4)	630 (2.9)	57 (2.5)	111 (2.7)
Hispanic	8667 (12.5)	3790 (9.9)	2746 (12.8)	301 (13.3)	561 (13.5)
White	33632 (48.6)	18500 (48.3)	11357 (52.8)	1249 (55.1)	2211 (53.4)
Other	1499 (2.2)	717 (1.9)	522 (2.4)	56 (2.5)	82 (2.0)
Multiracial	6279 (9.1)	3243 (8.5)	2072 (9.6)	207 (9.1)	405 (9.8)
Missing	15167 (21.9)	10289 (26.9)	3348 (15.6)	336 (14.8)	607 (14.6)
Public insurance	30527 (44.1)	14867 (38.8)	7997 (37.2)	799 (35.2)	1572 (37.9)
Smoking	887 (1.3)	641 (1.7)	247 (1.1)	2267 (100.0)	4142 (100.0)
Annual visit	31101 (58.8)	13741 (35.9)	21498 (100.0)	871 (38.4)	1623 (39.2)
Baseline caries risk					
Low	36038 (52.1)	23078 (60.2)	5045 (23.5)	1271 (56.0)	1880 (45.4)
Moderate	12642 (18.3)	6299 (16.4)	8185 (38.1)	548 (24.2)	1271 (30.7)
High	20532 (29.7)	8937 (23.3)	8268 (38.5)	449 (19.8)	993 (24.0)
Dental procedures*					
Evaluation	51680 (74.7)	28538 (74.5)	14712 (68.4)	1897 (83.6)	3530 (85.2)
Fluoride	31614 (45.7)	13623 (35.6)	17197 (80.0)	993 (43.8)	2161 (52.1)
Sealant	12408 (17.9)	6285 (16.4)	3088 (14.4)	0 (0.0)	903 (21.8)

*Procedures performed at the baseline of the follow-up (at time 0)
”Other” racial/ethnic group included American Indians, and Hawaiian/Pacific Islanders.

© 2024 Choi SE et al. *JAMA Network Open*.

eTable 3. Baseline Characteristics Before and After Near-Far Matching for Topical Fluoride

Receiving topical fluoride at least twice						
	Pre-matching			Post-matching		
	Encouraged (near) n=31804	Discouraged (far) n=28008	Abs St Dif	Encouraged (near) n=27504	Discouraged n=27504	Abs St Dif
Distance to clinic	2.68	17.75	1.12	2.68	17.82	1.17
Age at visit	10.78	11.16	0.07	10.93	10.95	0.00
Female	0.50	0.51	0.00	0.51	0.51	0.00
Male	0.50	0.49	0.00	0.49	0.49	0.00
Gender X	0.001	0.0006	0.02	0.0006	0.0006	0.00
Race_Asian	0.04	0.01	0.17	0.02	0.02	0.00
Race_Black	0.04	0.01	0.14	0.02	0.02	0.00
Race_Hispanic	0.11	0.10	0.03	0.11	0.11	0.00
Race_White	0.47	0.56	0.18	0.52	0.52	0.00
Race_Other	0.02	0.01	0.05	0.02	0.02	0.00
Race Multi	0.09	0.08	0.04	0.09	0.09	0.00
Race_Missing	0.22	0.21	0.03	0.21	0.21	0.00
Public insurance	0.43	0.30	0.27	0.37	0.37	0.01
Smoking	0.01	0.02	0.02	0.01	0.01	0.00
Oral evaluation	0.76	0.71	0.10	0.74	0.74	0.00
Sealant	0.15	0.15	0.02	0.15	0.15	0.00
Caries risk_Low	0.50	0.47	0.05	0.49	0.49	0.00
Caries risk_Mod	0.25	0.24	0.02	0.25	0.25	0.00
Caries risk_High	0.25	0.29	0.07	0.27	0.27	0.00
Annual visit	0.57	0.56	0.04	0.57	0.57	0.00

© 2024 Choi SE et al. *JAMA Network Open*.

eTable 4. Baseline Characteristics Before and After Near-Far Matching for Sealant

Receiving sealant by age 10						
	Encouraged (near) n=3553	Discouraged (far) n=2840	Abs St Dif	Encouraged (near) n=1855	Discouraged (far) n=1855	Abs St Dif
Distance to clinic	2.29	16.65	1.23	2.22	15.97	1.27
Female	0.50	0.51	0.01	0.50	0.50	0.00
Male	0.50	0.49	0.01	0.49	0.49	0.00
Gender X	0.0003	0.0001	0.04	0.00	0.00	NA
Race_Asian	0.05	0.02	0.17	0.02	0.02	0.00
Race_Black	0.04	0.01	0.16	0.02	0.02	0.00
Race_Hispanic	0.14	0.13	0.03	0.14	0.14	0.00
Race_White	0.50	0.58	0.16	0.57	0.57	0.00
Race_Other	0.02	0.02	0.03	0.02	0.02	0.00
Race Multi	0.10	0.09	0.01	0.09	0.09	0.00
Race_Missing	0.15	0.14	0.02	0.15	0.15	0.00
Public insurance	0.43	0.30	0.27	0.33	0.33	0.00
Oral evaluation	0.86	0.84	0.04	0.86	0.86	0.00
Topical Fluoride	0.50	0.49	0.02	0.49	0.49	0.00
Caries risk_Low	0.49	0.48	0.02	0.49	0.49	0.00
Caries risk_Mod	0.28	0.28	0.01	0.29	0.29	0.00
Caries risk_High	0.22	0.23	0.02	0.22	0.22	0.00
Annual visit	0.39	0.38	0.01	0.41	0.41	0.00

© 2024 Choi SE et al. *JAMA Network Open*.

eReferences.

1. Hernan MA, Robins JM. Using Big Data to Emulate a Target Trial When a Randomized Trial Is Not Available. *Am J Epidemiol.* 2016;183(8):758-764.
2. Hernan MA, Wang W, Leaf DE. Target Trial Emulation: A Framework for Causal Inference From Observational Data. *Jama.* 2022;328(24):2446-2447.
3. Centers for Medicare & Medicaid Services (CMS). Overview of the Dental and Oral Health Services Measures in the 2022 Child Core Set. https://www.medicaid.gov/quality-of-care/downloads/dentaloralhealth-ta-resource.pdf. Published 2022. Accessed Feb 5, 2023.
4. Iacus S, King G, Porro G. Causal Inference without Balance Checking: Coarsened Exact Matching. *Political Analysis.* 2012;20(1):1-24.
5. Uddin MJ, Groenwold RH, de Boer A, et al. Performance of instrumental variable methods in cohort and nested case-control studies: a simulation study. *Pharmacoepidemiol Drug Saf.* 2014;23(2):165-177.
6. Rigdon J, Baiocchi M, Basu S. Near-Far Matching in R: The nearfar Package. *J Stat Softw.* 2018;86(CS-5).
7. Baiocchi M, Small DS, Yang L, Polsky D, Groeneveld PW. Near/far matching: a study design approach to instrumental variables. *Health Serv Outcomes Res Methodol.* 2012;12(4):237-253.
8. Baiocchi MS, D.; Lorch, S. A.;& Rosenbaum, P. R. Building a Stronger Instrument in an Observational Study of Perinatal Care for Premature Infants. *J Am Stat Assoc.* 2010;105(492):1285-1296.
9. EA.; HDEIKKGS. Matching as Nonparametric Preprocessing for Reducing Model Dependence in Parametric Causal Inference. *Polit Anal.* 2007;15(03):199-236.
10. American Association of Pediatric Dentistry (AAPD). Caries-risk assessment and management for infants, children, and adolescents. *Pediatr Dent.* 2018;40(6):205-212.

© 2024 Choi SE et al. *JAMA Network Open*.
