# Supplementary material for: Quality Measure Adherence and Oral Health Outcomes in Children
Source: JAMA Netw Open. 2024 Jan 30;7(1):e2353861. doi: 10.1001/jamanetworkopen.2023.53861 (PMC10828912; doi:10.1001/jamanetworkopen.2023.53861)
Supplement: Supplement 2. — Data Sharing Statement [file jamanetwopen-e2353861-s002.pdf]

Data Sharing Statement

Choi. Quality Measure Adherence and Oral Health Outcomes in Children. *JAMA Netw Open*. Published January 30, 2024. doi:10.1001/jamanetworkopen.2023.53861

Data
Data available: No

Additional Information
Explanation for why data not available: The data that support the findings of this study were accessed under a data use agreement and are not publicly available.
